# Plasma biomarkers predict amyloid pathology in cognitively normal monozygotic twins after 10 years

**DOI:** 10.1093/braincomms/fcad024

**Published:** 2023-02-04

**Authors:** Anouk den Braber, Inge M W Verberk, Jori Tomassen, Ben den Dulk, Erik Stoops, Jeffrey L Dage, Lyduine E Collij, Frederik Barkhof, Gonneke Willemsen, Michel G Nivard, Bart N M van Berckel, Philip Scheltens, Pieter Jelle Visser, Eco J C de Geus, Charlotte E Teunissen

**Affiliations:** Alzheimer Center Amsterdam, Neurology, Vrije Universiteit Amsterdam, Amsterdam UMC location VUmc, Amsterdam, The Netherlands; Amsterdam Neuroscience, Neurodegeneration, Amsterdam, The Netherlands; Department of Biological Psychology, Vrije Universiteit Amsterdam, Amsterdam, The Netherlands; Amsterdam Neuroscience, Neurodegeneration, Amsterdam, The Netherlands; Neurochemistry Laboratory Department of Clinical Chemistry, Vrije Universiteit Amsterdam, Amsterdam UMC location VUmc, Amsterdam, The Netherlands; Alzheimer Center Amsterdam, Neurology, Vrije Universiteit Amsterdam, Amsterdam UMC location VUmc, Amsterdam, The Netherlands; Amsterdam Neuroscience, Neurodegeneration, Amsterdam, The Netherlands; Amsterdam Neuroscience, Neurodegeneration, Amsterdam, The Netherlands; Neurochemistry Laboratory Department of Clinical Chemistry, Vrije Universiteit Amsterdam, Amsterdam UMC location VUmc, Amsterdam, The Netherlands; ADx NeuroSciences, Ghent, Belgium; Stark Neurosciences Research Institute, Indiana University School of Medicine, Indianapolis, USA; Department of Radiology & Nuclear Medicine, Vrije Universiteit Amsterdam, Amsterdam UMC location VUmc, Amsterdam, The Netherlands; Amsterdam Neuroscience, Brain Imaging, Amsterdam, The Netherlands; Department of Radiology & Nuclear Medicine, Vrije Universiteit Amsterdam, Amsterdam UMC location VUmc, Amsterdam, The Netherlands; Amsterdam Neuroscience, Brain Imaging, Amsterdam, The Netherlands; UCL Institute of Neurology, London, UK; Department of Biological Psychology, Vrije Universiteit Amsterdam, Amsterdam, The Netherlands; Department of Biological Psychology, Vrije Universiteit Amsterdam, Amsterdam, The Netherlands; Department of Radiology & Nuclear Medicine, Vrije Universiteit Amsterdam, Amsterdam UMC location VUmc, Amsterdam, The Netherlands; Amsterdam Neuroscience, Brain Imaging, Amsterdam, The Netherlands; Alzheimer Center Amsterdam, Neurology, Vrije Universiteit Amsterdam, Amsterdam UMC location VUmc, Amsterdam, The Netherlands; Amsterdam Neuroscience, Neurodegeneration, Amsterdam, The Netherlands; Alzheimer Center Amsterdam, Neurology, Vrije Universiteit Amsterdam, Amsterdam UMC location VUmc, Amsterdam, The Netherlands; Amsterdam Neuroscience, Neurodegeneration, Amsterdam, The Netherlands; Alzheimer Center Limburg, School for Mental Health and Neuroscience, Maastricht University, Maastricht, The Netherlands; Department of Neurobiology, Care Sciences and Society, Division of Neurogeriatrics, Karolinska Institutet, Stockholm, Sweden; Department of Biological Psychology, Vrije Universiteit Amsterdam, Amsterdam, The Netherlands; Amsterdam Neuroscience, Neurodegeneration, Amsterdam, The Netherlands; Neurochemistry Laboratory Department of Clinical Chemistry, Vrije Universiteit Amsterdam, Amsterdam UMC location VUmc, Amsterdam, The Netherlands

**Keywords:** amyloid pathology, monozygotic twins, plasma biomarkers, confounding, longitudinal dynamics

## Abstract

Blood-based biomarkers could prove useful to predict Alzheimer’s disease core pathologies in advance of clinical symptoms. Implementation of such biomarkers requires a solid understanding of their long-term dynamics and the contribution of confounding to their association with Alzheimer’s disease pathology. Here we assess the value of plasma amyloid-β_1-42/1-40_, phosphorylated-tau181 and glial fibrillary acidic protein to detect early Alzheimer’s disease pathology, accounting for confounding by genetic and early environmental factors. Participants were 200 monozygotic twins, aged ≥60 years with normal cognition from the european medical information framework for Alzheimer's disease study. All twins had amyloid-β status and plasma samples available at study enrolment. For 80 twins, additional plasma samples were available that had been collected approximately 10 years prior to amyloid-β status assessment. Single-molecule array assays were applied to measure amyloid-β_1-42/1-40_, phosphorylated-tau181 and glial fibrillary acidic protein. Predictive value of and longitudinal change in these biomarkers were assessed using receiver operating characteristic curve analysis and linear mixed models. Amyloid pathology could be predicted using blood-based biomarkers obtained at the time of amyloid status assessment (amyloid-β_1-42/1-40_: area under the curve = 0.65, *P* = 0.01; phosphorylated-tau181: area under the curve = 0.84, *P* < 0.001; glial fibrillary acidic protein: area under the curve = 0.74, *P* < 0.001), as well as using those obtained 10 years prior to amyloid status assessment (amyloid-β_1-42/1-40_: area under the curve = 0.69, *P* = 0.03; phosphorylated-tau181: area under the curve = 0.92, *P* < 0.001; glial fibrillary acidic protein: area under the curve = 0.84, *P* < 0.001). Longitudinally, amyloid-β_1-42/1-40_ levels decreased [*β* (SE) = −0.12 (0.01), *P* < 0.001] and phosphorylated-tau181 levels increased [*β* (SE) = 0.02 (0.01), *P* = 0.004]. Amyloid-β-positive individuals showed a steeper increase in phosphorylated-tau181 compared with amyloid-β-negative individuals [*β* (SE) = 0.06 (0.02), *P* = 0.004]. Also amyloid-β-positive individuals tended to show a steeper increase in glial fibrillary acidic protein [*β* (SE) = 0.04 (0.02), *P* = 0.07]. Within monozygotic twin pairs, those with higher plasma phosphorylated-tau181 and lower amyloid-β_1-42/1-40_ levels were more likely to be amyloid-β positive [*β* (SE) = 0.95 (0.26), *P* < 0.001; *β* (SE) = −0.28 (0.14), *P* < 0.05] indicating minimal contribution of confounding by genetic and early environmental factors. Our data support the use of amyloid-β_1-42/1-40_, phosphorylated-tau181 and glial fibrillary acidic protein as screening tools for Alzheimer’s disease pathology in the normal aging population, which is of importance for enrolment of high-risk subjects in secondary, or even primary, prevention trials. Furthermore, these markers show potential as low-invasive monitoring tool of disease progression and possibly treatment effects in clinical trials.

## Teaser

den Braber *et al*. report that plasma levels of amyloid-β_1-42/1-40_, p-tau181 and GFAP can be used to predict amyloid-β pathology in older cognitively unimpaired individuals, as long as 10 years before the presence of amyloid-β pathology, indicating the potential of these markers as early diagnostic tools in the normal aging population.

## Graphical Abstract

**Graphical Abstract:**
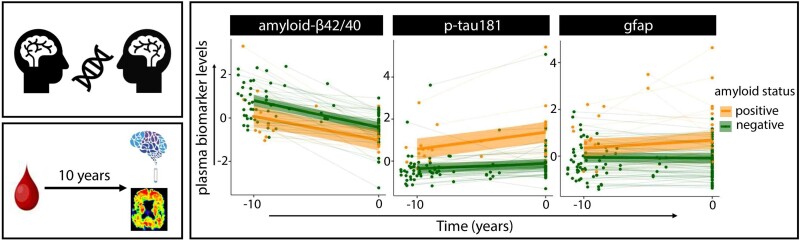

## Introduction

The number of Alzheimer’s disease patients is growing rapidly and is expected to almost double every 20 years, thereby becoming one of the major future causes of death.^1^ To be able to stop this rapid progression, we need to develop treatment strategies to slow or stop the development of Alzheimer’s disease. But in order to get there, a precise and early diagnosis of Alzheimer’s disease is a requirement.

Nowadays, the earliest pathological hallmarks such as amyloid-β and tau accumulation can be accurately measured by means of protein quantification in cerebrospinal fluid (CSF) and with positron emission tomography (PET) imaging and are as such key in the biological diagnosis of Alzheimer’s disease.^2^ However, measurement of these biomarkers is expensive or invasive and not suitable for broad implementation in the elderly population. The development of minimally invasive blood biomarker panels with comparable clinical performance to CSF and PET has therefore been the focus of investigation for several decades. Recent studies showed blood-based biomarkers to be useful to identify the core pathologies in Alzheimer’s disease, such as amyloid-β and phosphorylated tau (p-tau) deposition, neurodegeneration (using neurofilament light) and astrocyte re-activity [using glial fibrillary acidic protein (GFAP)].^3–13^ The combination of these markers showed to improve prediction of amyloid-β status on PET.^3^ In addition, these biomarkers were able to predict neurodegeneration, cognitive change and future Alzheimer’s disease dementia in cognitively unimpaired elderly.^14–19^ Also, longitudinal changes in plasma p-tau in the earliest stages of Alzheimer’s disease depended on amyloid-β status and correlated with longitudinal worsening of cognition and brain atrophy.^15,16^

Before implementation of these blood-based biomarkers of Alzheimer’s disease pathology in trials targeting heterogeneous elderly populations, it is relevant to understand the effects of possible confounding factors, such as age, lifestyle and genetic variation, to prevent misdiagnosis or inappropriate selection of participants at risk for developing Alzheimer’s disease for clinical trials.^20^ In addition, the dynamics of these blood-based markers over long time intervals need to be established in cognitively unimpaired individuals before they can be implemented in clinical practice.

Within this study, we aim to investigate the potential of blood-based biomarkers amyloid-β_1-42/1-40_, p-tau181 and GFAP as early amyloid-β pathology detection tools in the normal aging population, taking confounding and longitudinal dynamics into consideration. We do this by assessing both the cross-sectional and prognostic values of these markers for predicting amyloid-β pathology in cognitively unimpaired elderly monozygotic twins, using plasma samples that were collected at the time of amyloid-β status assessment, as well as 10 years prior. In addition, longitudinal change in these blood-based biomarkers and their interaction with amyloid-β status are assessed. We hypothesize these markers to predict amyloid-β status with high accuracy even in cognitively unimpaired individuals. The possible influence of confounding factors is examined by making use of our unique twin population. Due to the highly matched nature of our sample of monozygotic twin pairs that share not just age and sex but all of their genome as well as a substantial shared environmental history, they offer the unique opportunity to study the predictive value of blood-based biomarkers for Alzheimer’s disease pathology, while controlling for genetic and shared environmental confounding which is not possible in general population studies.^21,22^ Interestingly, although all were cognitively healthy, we previously observed that 14 of the 94 monozygotic twin pairs were discordant (one twin affected and the co-twin unaffected) for having amyloid-β pathology (as measured with PET).^23^ Based on the hypothesis that the blood-based biomarkers can sensitively detect amyloid-β pathology, we expect these twin differences in amyloid-β status to also be reflected in their blood-based biomarker levels.

## Materials and methods

### Participants

We selected 200 monozygotic twins (97 complete pairs), aged ≥60 years from the ongoing Amsterdam sub-study of the european medical information framework for Alzheimer's disease (EMIF-AD PreclinAD) cohort. All had normal cognition, based on a global Clinical Dementia Rating score of 0, with a score of 0 on the memory sub-domain and delayed recall score >−1.5 SD of demographic-adjusted normative data on the Consortium to Establish a Registry for Alzheimer’s Disease 10-word list. Exclusion criteria were any significant neurologic, systemic or psychiatric disorder that could cause cognitive impairment.^24^ All twins had amyloid-β status and plasma samples available at the timepoint of study enrolment. In addition, from 80 twins, plasma samples were available that had been collected approximately 10 years [median (interquartile range) = −9.7 (2)] prior to amyloid-β status assessment as part of the Netherlands Twin Register biobank study.^25^ Twin zygosity was confirmed at baseline by buccal cell DNA analysis. All participants gave written informed consent.

### Amyloid-β status assessment

During the EMIF-AD PreclinAD baseline visit, twins underwent dynamic dual-time window [^18^F]flutemetamol PET and CSF was obtained by lumbar puncture on the same day as blood collection.^24^ Amyloid-β status was based on PET (*n* = 195) or CSF measures (*n* = 126). For PET status assessment, we classified twins as amyloid-β positive or negative by visual reading of the [^18^F]flutemetamol scans. Rating was performed on the parametric non-displaceable binding potential images by three readers trained according to General Electric Healthcare guidelines. In case of inconsistency, the consensus rating of two readers was used.

For CSF status assessment, first levels of amyloid-β_1-40_ and amyloid-β_1-42_ were measured with kits from the same batch according to manufacturer instructions (Euroimmun).^24^ Next, we used a Gaussian mixture modelling to determine a cut-off for CSF amyloid-β_1-42/1-40_ ratio. Two distributions showed the best fit, and we used the point of intersection between these distributions as a cut-off to indicate abnormality (CSF amyloid-β_1-42/1-40_ < 0.066 pg/ml). A subject was marked amyloid-β positive when either CSF or PET status was positive (discordance in PET versus CSF amyloid-β positivity; *n* = 8 in the total sample of 200 subjects and *n* = 1 in the sub-sample of 80 subjects with 10-year-old blood samples available).

### Blood collection

#### EMIF PreclinAD study

During the EMIF-AD PreclinAD baseline visit, plasma samples were collected at the Amsterdam University Medical Centre, location VUmc, through venipuncture in the morning, after 2 h of fasting. Ethylenediaminetetraacetic acid tubes with anti-coagulated whole blood were centrifuged at 1300–2000 × g for 10 min within 2 h, and plasma and remaining buffy coat were aliquoted in aliquots of 0.25–0.5 ml and immediately stored at −80°C until analysis.^24,26^

#### Netherlands Twin Register biobank study

Prior plasma samples had been collected 10 years earlier at the participants’ home through venipuncture in the morning after overnight fasting. Ethylenediaminetetraacetic acid tubes with anti-coagulated whole blood were stored in melting ice during transport and centrifuged at 2000 × g at 4°C for 20 min within 6 h after sampling. Plasma was aliquoted in aliquots of 0.5 ml, snap frozen in dry ice and stored at −30°C.^25^

### Plasma biomarker assessment

Plasma biomarker assessment was performed at the Amsterdam University Medical Centre, location VUmc, Neurochemistry laboratory. Samples were thawed at room temperature and centrifuged at 10 000 g for 10 min. Subsequently, plasma was analysed using the automated single-molecule array HDx analyzer using the in-house developed AMYBLOOD assay (Amsterdam UMC, ADx NeuroSciences) that simultaneously measures plasma concentrations amyloid-β_1-42_ and amyloid-β_1-40_. GFAP was measured with the commercially available single-molecule array assay (Quanterix corporation, Billerica, USA) according to the kit instructions, and p-tau181 was measured using a single-molecule array prototype two-step assay with AT270 (Thermo Fisher Scientific) as the capture and LRL (Eli Lilly and Company) as the detector antibody as described previously.^26^ Analyses were performed in duplicates, measuring longitudinal samples of twin pairs in the same run, using a 1:4 automated dilution protocol, according to the manufacturer’s instructions. The assays were in-house analytically validated according to a standardized international consensus protocol.^27^

### Statistical analysis

Statistical analyses were performed in SPSS version 25 and R version 4.1.0.

Receiver operating characteristic curve analysis was performed to determine the value of plasma amyloid-β_1-42/1-40_, p-tau181 and GFAP in predicting amyloid-β status, both cross-sectionally and using the data from samples collected 10 years prior to amyloid-β status assessment. Youden’s cut-offs were calculated as the maximum sum of sensitivity and specificity.

Linear mixed models, adjusted for age, sex and Apolipoproteïne E (*ApoE)*-ε4 carriership, were applied to assess longitudinal change in plasma biomarker levels, using time and the interaction between time and amyloid-β status as a predictor. Within twin-pair correlations for plasma amyloid-β_1-42/1-40_, p-tau181 and GFAP levels, adjusted for age and sex, were assessed to estimate similarities in monozygotic twins for these markers. To test whether observed relations between plasma biomarker levels and amyloid-β status were influenced by confounding, we performed logistic regression analyses using generalized estimating equations, with family biomarker level [(plasma biomarker level twin 1 + plasma biomarker level twin 2)/2] and biomarker level delta (personal plasma biomarker level − family biomarker level) as predictors and amyloid-β status as outcome measure. Standard errors were clustered within family. The regression coefficient for biomarker level delta tests whether twins with higher plasma p-tau181 and GFAP and lower amyloid-β_1-42/1-40_ levels compared to their co-twin, corrected for all shared family factors (age, sex, genome, shared environmental history), are also more likely to be amyloid-β positive. Significant associations between biomarker level delta and the outcome indicate observed associations to be minimally affected by genetic and shared environmental confounding.^28^

### Data availability

Data will be available (including data dictionaries and study protocol), immediately following publication, for *bona fide* researchers who provide a methodologically sound research proposal. Data sharing requests for such research proposals should be directed to ntr.datamanagement.fgb@vu.nl (see https://tweelingenregister.vu.nl/information_for_researchers/working-with-ntr-data for NTR data sharing procedures). To gain access, data requestors will need to sign a data access agreement.

## Results

### Population characteristics per sample collection timepoint

Population characteristics are presented in **Table 1**.

**Table 1:** Sample characteristics.

	Total sample	Amyloid−	Amyloid+
Samples collected at the time of amyloid status assessment			
*N* (%)	200	167 (83.5)	33 (16.5)
Age in years, mean (SD)	70.5 (7.5)	69.6 (7.3)	75.3 (7.1)
Female gender, *n* (%)	115 (57.5)	93 (55.7)	22 (66.7)
ApoE-ε4 carriers, *n* (%)	67 (33.5)	51 (30.5)	16 (48.5)
Plasma p-tau181 pg/ml, mean (SD) (*n* = 186)	6.4 (2.6)	5.9 (2.0)	9.1 (3.6)
Plasma GFAP pg/ml, mean (SD) (*n* = 200)	153.1 (89.5)	141.2 (77.2)	213.8 (119.7)
Plasma Aβ_42/40_ pg/ml, mean (SD) (*n* = 181)	0.24 (0.04)	0.24 (0.04)	0.22 (0.05)
Samples collected 10 years prior to amyloid status assessment			
N (%)	80	66 (82.5)	14 (17.5)
Age in years, mean (SD)	59 (6.3)	57.7 (5.3)	64.7 (7.5)
Female gender, *n* (%)	44 (55)	35 (53.0)	9 (64.3)
ApoE-ε4 carriers, *n* (%)	32 (40)	23 (34.8)	9 (64.3)
Plasma p-tau181 pg/ml, mean (SD) (*n* = 78)	5.5 (2.5)	4.9 (2.1)	7.9 (2.3)
Plasma GFAP pg/ml, mean (SD) (*n* = 80)	140.9 (67.6)	123.7 (49.6)	221.9 (83.1)
Plasma Aβ_42/40_ pg/ml, mean (SD) (*n* = 73)	0.29 (0.05)	0.30 (0.04)	0.27 (0.06)

Amyloid groups were based on PET visual read and/or CSF Aβ_42/40_ ratio using cutoff <0.066. Aβ: amyloid beta; GFAP: glial fibrillary acidic protein.

#### Samples collected at the time of amyloid-β status assessment

The 200 cognitively unimpaired twins from whom blood samples and amyloid-β status were obtained as part of the EMIF-AD PreclinAD study were on average 70.5 years old, 57.5% was female, 33.5% carried at least one *ApoE*-ε4 allele and 16.5% showed amyloid-β pathology on PET/CSF. Plasma GFAP levels were available for all participants. Due to technical analysis failure, plasma p-tau181 and amyloid-β_1-42/1-40_ levels were missing for respectively 14 (7%) and 19 (9.5%) participants.

#### Samples collected 10 years prior to amyloid-β status assessment

The 80 twins with plasma samples available that were collected with a median (interquartile range) of 9.7 (2) years prior to amyloid-β status assessment were on average 59 years old at the time of blood sampling. Within this sub-cohort, 55% was female, 40% carried at least one *ApoE*-ε4 allele and 17.5% was amyloid-β positive on PET/CSF 10 years later. For this sample, plasma GFAP levels could be analysed in all participants, whereas measurement of plasma p-tau181 failed in two cases (2.5%) and of amyloid-β_1-42/1-40_ in seven cases (9%).

### Receiver operating characteristic curve analyses

#### Samples collected at the time of amyloid-β status assessment

**Figure 1:**
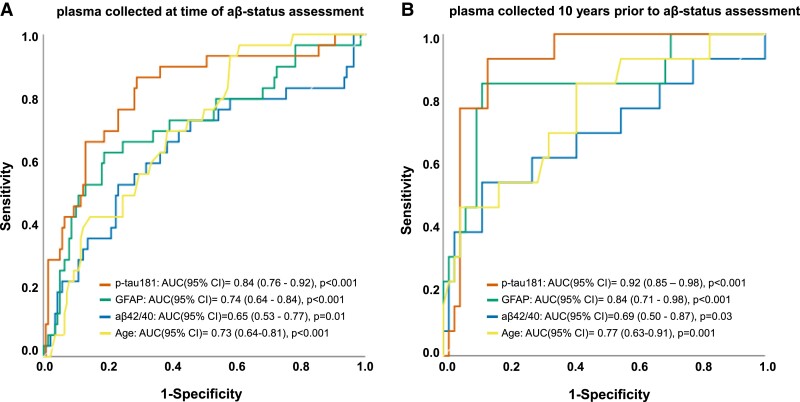
**Receiver operating characteristic curve analyses.** Receiver operating characteristic area under the curve analyses predicting amyloid pathology using plasma amyloid-β_1-42/1-40_ (*n* = 181), p-tau181 (*n* = 186) and GFAP (*n* = 200) levels obtained at the time of amyloid-β status assessment (**A**) or plasma amyloid-β1-42/1-40 (*n* = 73), p-tau181 (*n* = 78) and GFAP (*n* = 80) levels obtained 10 years prior to amyloid-β status assessment (**B**). AUC: area under the curve; CI: confidence interval.

Receiver operating characteristic curve analyses in the total cohort showed all plasma markers, obtained at time of amyloid-β status assessment, to predict amyloid-β status. Plasma p-tau181 showed the highest area under the curve [0.84 (0.76–0.92)], sensitivity (0.88) and negative predictive value (0.97), and plasma GFAP showed the highest specificity (0.83) (Fig. 1A; Supplementary Table 1). Comparing areas under the curve using DeLong’s test showed plasma p-tau181 to significantly outperform plasma GFAP (*Z* = 2.1, *P* = 0.04), plasma amyloid-β_1-42/1-40_ (*Z* = 2.4, *P* = 0.02) and age (*Z* = 2.3, *P* = 0.03) in discriminating amyloid-β status. Combining the markers did not significantly improve discrimination of amyloid-β status (*Z* =−0.16, *P* = 0.87). Repeating the analyses in the sub-cohort for which all markers were available (*n* = 168) did not change results.

#### Samples collected 10 years prior to amyloid-β status assessment

Plasma markers obtained 10 years prior to amyloid-β status assessment were also able to discriminate amyloid-β positive from negative individuals (Fig. 1B; Supplementary Table 2). Plasma p-tau181 showed the highest area under the curve [0.92 (0.85–0.98)], sensitivity (0.93) and negative predictive value (0.98). DeLong’s test indicated plasma p-tau181 to significantly outperform plasma amyloid-β_1-42/1-40_ (*Z* = 2.2, *P* = 0.03) and, at trend level, age (*Z* = 1.7, *P* = 0.08) in discriminating amyloid-β status, but not GFAP. Repeating the analyses in the sub-cohort for which all markers were available (*n* = 71) did not change results.

### Longitudinal change in blood-based biomarker levels

**Figure 2:**
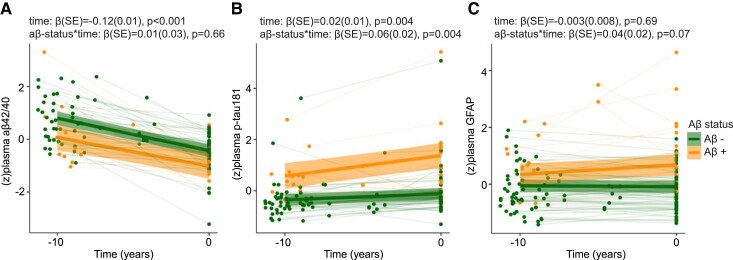
**Longitudinal change in blood-based biomarker levels.** Shown are spaghetti plots of z-transformed plasma biomarkers (*y*-axis) against time (*x*-axis) for individuals with and without amyloid pathology. We superimposed average slopes per group derived from linear mixed models adjusted for age, sex and ApoE-ε4 carriership. Reported from the linear mixed models are the fixed effects of time and aβ-status*time. (**A**) Plasma amyloid-β_1-42/1-40_ (*n* = 62). (**B**) Plasma p-tau181 (*n* = 74). (**C**) Plasma GFAP (*n* = 80). Orange = amyloid-β-positive individuals. Green = amyloid-β-negative individuals. Time (years) 0 = time of amyloid status assessment.

Linear mixed models showed a significant effect of amyloid-β status on all blood-based biomarker levels (Fig. 2, **Table 2**). Longitudinally, we observed an overall decrease in plasma amyloid-β_1-42/1-40_ [*β* (SE) = −0.12 (0.01), *P* < 0.001, *P*_FDR_ < 0.001] (Fig. 2A) and an increase in plasma p-tau181 levels over time [*β* (SE) = 0.02 (0.01), *P* = 0.004, *P*_FDR_ = 0.006] (Fig. 2B), but no significant change for GFAP [*β* (SE) = −0.003 (0.008), *P* = 0.69, *P*_FDR_ = 0.69] (Fig. 2C). The observed decrease in plasma amyloid-β_1-42/1-40_ levels was highly similar for amyloid-β-positive and amyloid-β-negative individuals (Fig. 2A). Interestingly, amyloid-β-positive individuals showed a steeper increase in plasma p-tau181 levels over 10 years compared to amyloid-β-negative individuals [*β* (SE) = 0.06 (0.02), *P* = 0.004, *P*_FDR_ = 0.01] (Fig. 2B). Also, at trend level, amyloid-β-positive individuals showed a steeper increase in plasma GFAP levels over 10 years compared to amyloid-β-negative individuals [*β* (SE) = 0.04 (0.02), *P* = 0.07, *P*_FDR_ = 0.09] (Fig. 2C).

**Table 2:** Longitudinal change in blood-based biomarkers.

	(z)plasma aβ_1-42/1-40_ (*n* = 62; 12 aβ+, 50 aβ−)			(z)plasma p-tau181 (*n* = 74; 14 aβ+, 60 aβ−)			(z)plasma GFAP (*n* = 80; 14 aβ+, 66 aβ−)		
Fixed effects	Beta (SE)	*P*	*P*_FDR_	Beta (SE)	*P*	*P*_FDR_	Beta (SE)	*P*	*P*_FDR_
Amyloid status	−0.60 (0.28)	0.04	0.04	1.49 (0.29)	<0.001	<0.001	0.77 (0.20)	<0.001	<0.001
Time	−0.12 (0.01)	<0.001	<0.001	0.02 (0.01)	0.004	0.006	−0.003 (0.008)	0.69	0.69
Amyloid status × time	0.01 (0.03)	0.66	0.66	0.06 (0.02)	0.004	0.01	0.04 (0.02)	0.07	0.09

### Twin-specific analyses

#### Twin correlations for blood-based biomarker levels and slopes

**Figure 3:**
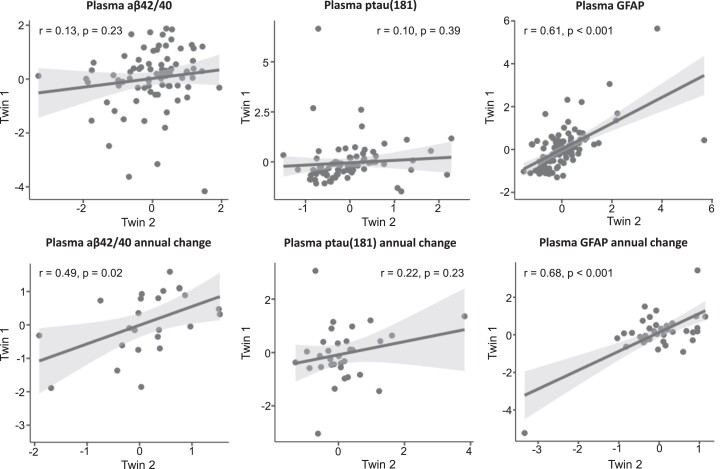
**Monozygotic twin correlations.** Within monozygotic twin-pair correlations (Pearson) for plasma biomarker levels at the time of amyloid-β status assessment (*top* row: amyloid-β_1-42/1-40_, *n*_pairs_ = 81; p-tau181, *n*_pairs_ = 85; GFAP, n_pairs_ = 97) and for annual change in blood-based biomarker levels (*bottom* row: amyloid-β_1-42/1-40_, *n*_pairs_ = 24; p-tau181, *n*_pairs_ = 32; GFAP, *n*_pairs_ = 37), adjusted for age and sex. Plasma levels were scaled prior to analyses.

Monozygotic twin pairs were highly similar for plasma GFAP, both for levels obtained at time of plasma amyloid-β status assessment (*r* = 0.61, *P* < 0.001, *P*_FDR_ < 0.001) and for their annual change in plasma GFAP levels (*r* = 0.68, *P* < 0.001, *P*_FDR_ ≤ 0.001) (Fig. 3), indicating genetic factors to play an important role in astrocyte activation. A moderate twin correlation was found for the annual change in plasma amyloid-β_1-42/1-40_ levels (*r* = 0.49, *P* = 0.02, *P*_FDR_ = 0.02), suggesting that the natural course of plasma amyloid-β_1-42/1-40_ during aging is influenced by genetic factors. No significant within-pair correlations were observed for plasma amyloid-β_1-42/1-40_ and plasma p-tau181 levels at the time of amyloid-β status assessment, and annual change in plasma p-tau181 levels, suggesting these processes to be influenced by environmental factors that are unique to an individual, which also include measurement error.

#### Association plasma biomarkers and amyloid-β status controlled for genetic and familial confounding

Our logistic regression analyses, correcting for shared family factors, showed that even though monozygotic twins have the same age and sex and share 100% of their genetic material as well as a substantial shared environmental history, those with higher plasma p-tau181 levels are more likely to be amyloid-β positive [*β* (SE) = 0.95 (0.26), *P* < 0.001, *P*_FDR_ ≤ 0.001]. Similar effects were observed for amyloid-β_1-42/1-40_, where the twins with lower levels of plasma amyloid-β_1-42/1-40_ within a pair were more likely to be amyloid-β positive [*β* (SE) = −0.28 (0.14), *P* < 0.05, *P*_FDR_ = 0.07]. This indicates that the observed relation between plasma p-tau181 and amyloid-β_1-42/1-40_ and brain amyloid-β pathology is minimally affected by age, sex or genetic and shared environmental confounding. For GFAP, the direction of effect was the same (GFAP levels were higher in the amyloid-β-positive twin), but not significant [*β* (SE) = 0.08 (0.16), *P* = 0.60, *P*_FDR_ = 0.60].

## Discussion

Our longitudinal study in a cohort of monozygotic twins shows that plasma levels of amyloid-β_1-42/1-40_, p-tau181 and GFAP can be used to predict amyloid-β pathology in elderly cognitively unimpaired individuals, even as long as 10 years before we assessed the presence of amyloid-β pathology, indicating the potential of these markers as early diagnostic tools in the normal aging population. Also, within this 10-year time frame, we found amyloid-β-dependent longitudinal increases in plasma p-tau181, and to a lesser extent GFAP, indicating these markers to be potentially useful for disease monitoring in very early disease stages.

### p-tau181

The highest discriminative accuracy was observed for plasma p-tau181. Similar high area under the curve, sensitivity and specificity have been previously found for this marker in predicting amyloid-β status in pre-clinical cohorts.^4,5^ Also, in line with our findings, Mattsson *et al.*^16^ observed amyloid-β-dependent longitudinal increases in plasma p-tau which were correlated with longitudinal worsening of cognition and brain atrophy. Our findings that these Alzheimer’s disease pathology-dependent increases were already detectable in the 10-year time frame prior to CSF/PET-confirmed amyloid-β pathology in cognitively still unimpaired individuals suggest that Alzheimer’s disease-related changes in plasma p-tau levels are already present in very early stages of Alzheimer’s disease development. We additionally show that controlling for age, sex and all the shared early environmental and genetic influences within monozygotic twins did not change the discriminatory effect of plasma p-tau181 on amyloid-β status. This finding is compatible with a causal link between plasma p-tau metabolism and brain amyloid-β accumulation as previously suggested.^5^ Although the mechanisms underlying this putative causality need further study, our data underscore the usefulness of plasma p-tau as a very early diagnostic and prognostic biomarker of Alzheimer’s disease pathology.

### GFAP

We also observed high discriminative accuracy for plasma GFAP. Interestingly, levels of GFAP remained largely stable over 10 years’ time in cognitively unimpaired individuals that were amyloid-β negative, whereas subtle longitudinal increases were observed in amyloid-β-positive subjects. These results replicate previous findings showing the diagnostic value of this biomarker as well as its monitoring potential.^3,17,29–31^ GFAP is a marker of astrocyte activation, the brain’s response to neuronal injury, including amyloid-β and tau pathology.^32–34^ Although the exact role of astrocyte activation in early Alzheimer’s disease pathology needs further study, it has been suggested that reactive astrocytes may act as initiators and/or modulators of early Alzheimer’s disease pathology and progression.^32–35^ Our findings that amyloid-β-positive individuals show higher GFAP levels compared to amyloid-β-negative individuals and subtle increases in the early pre-dementia stage support this hypothesis and suggest increases in plasma GFAP levels to be a direct effect of brain amyloid-β pathology. Resemblance of monozygotic twins for plasma GFAP levels was high, indicating that the process of astrocytosis may be, to a large extent, genetically determined. Furthermore, our twin-specific analysis showed plasma GFAP levels not to be significantly higher in the amyloid-β-positive twin, which suggests the observed relation between amyloid-β pathology and plasma GFAP reflects genetic confounding. Hence, part of the genetic factors that influence plasma levels of GFAP may also (independently) influence amyloid-β pathology in the brain. However, we want to be cautious in drawing these conclusions. It might be that in the earliest stages of Alzheimer’s disease, differences between monozygotic twins in a highly heritable trait go undetected.

### Amyloid-β_1-42/1-40_

In line with previous studies, we found that levels of plasma amyloid-β_1-42/1-40_ were lower in amyloid-β-positive compared to amyloid-β-negative individuals.^10–12,29,36^ Although this marker significantly predicted amyloid-β status in our cognitively unimpaired cohort, even using plasma obtained 10 years prior to status assessment, it showed lower discriminative accuracy compared to p-tau181 and GFAP, probably due to the small relative changes and measurement error on single-molecule array.^13^ In fact, it did not significantly outperform age as a predictor of amyloid-β status. This is in agreement with previous studies showing that classification performance of this marker was better in subjects with clinical dementia than those in pro-dromal and pre-clinical stages and improved when combined with age, *ApoE*-ε4 carriership and GFAP.^13^ The observed amyloid-β-independent longitudinal decreases might indicate these decreases strongly depend on age, further complicating the clinical use of this biomarker for prognostic purposes.

This study has a number of limitations. First, the amyloid-β status at the time of first blood collection was unknown, limiting the ability to draw conclusions on timing of events. Second, the number of twins with longitudinal blood samples available was relatively low. Third, no fraternal twins were measured, which precludes separation of additive and non-additive genetic effects or detection of the role of the shared early environment on these biomarkers. These results therefore need further replication in larger longitudinal (twin) cohorts with longitudinal samples stored for prolonged periods.

Of note, our data indicates single-molecule array assays to robustly and accurately measure plasma amyloid-β_1-42/1-40_, p-tau181 and GFAP even when already stored for 10 years under variable conditions. This offers enormous potential for long-term stored plasma samples in epidemiological studies. Such large cohorts with available plasma data are not only useful for replication purposes, but also they offer many promising research opportunities, including identification of contributors to Alzheimer’s disease risk and confounding factors .^37^

Taken together, our results indicate changes in plasma amyloid-β_1-42/1-40_, p-tau181 and GFAP levels to be present already in very early stages of Alzheimer’s disease development, and that longitudinal, amyloid-β status-dependent changes in plasma p-tau181 and GFAP reflect disease progression in this pre-clinical stage. Thereby, these blood-based biomarkers, and especially p-tau181 and GFAP, have great potential as early amyloid-β pathology pre-screening tools in the normal aging population which can be of great benefit for the enrolment of high-risk subjects in behavioural and pharmacological secondary, or even primary, prevention trials and for monitoring of disease progression and treatment effects in clinical trials.

## Supplementary Material

fcad024_Supplementary_Data

## Abbreviations

GFAP =: glial fibrillary acidic protein
NPV =: negative predictive value
PPV =: positive predictive value
