# Supplementary material for: Plasma biomarkers predict amyloid pathology in cognitively normal monozygotic twins after 10 years
Source: Brain Commun. 2023 Feb 4;5(1):fcad024. doi: 10.1093/braincomms/fcad024 (PMC9942541; doi:10.1093/braincomms/fcad024)
Supplement: fcad024_Supplementary_Data [file fcad024_supplementary_data.pdf]

Supplementary material

Supplementary Table 1 | Receiver operating characteristic curves predicting amyloid pathology using blood-based biomarkers levels obtained at time of amyloid-β status assessment.

**Predictor** (n aβ+/aβ-)	**AUC (95% CI)**	**p-value**	**p_{FDR}**	**Youden's cut-point**	**Sensitivity**	**Specificity**	**PPV**	**NPV**
Plasma $a\beta_{1-42/1-40}$ (31/150)	0·65 (0·53 - 0·77)	0·01	0·01	0·22	0·55	0·75	0·31	0·89
Plasma p-tau181 (32/154)	0·84 (0·76 - 0·92)	<0·001	<0·001	6·4 pg/ml	0·88	0·73	0·41	0·97
Plasma GFAP (33/167)	0·74 (0·64 - 0·84)	<0·001	<0·001	175·3 pg/ml	0·64	0·83	0·42	0·92
Age (33/167)	0·73 (0·64-0·81)	<0·001	<0·001		0·94	0·41	0·24	0·97

AUC: area under curve; CI: confidence interval; p_{FDR}: false discovery rate adjusted p value; PPV: positive predictive value; NPV: negative predictive value. Youden's cut-point is at the coordinates of the ROC curve where a maximum sum of sensitivity and specificity is reached.

Supplementary Table 2 | Receiver operating characteristic curves predicting amyloid pathology using blood-based biomarkers levels obtained 10 years prior to amyloid-β status assessment.

**Predictor** (n aβ+/aβ-)	**AUC (95% CI)**	**p-value**	**p_{FDR}**	**Youden's cut-point**	**Sensitivity**	**Specificity**	**PPV**	**NPV**
Plasma $a\beta_{1-42/1-40}$ (13/60)	0·69 (0·50 - 0·87)	0·03	0·03	0·25	0·54	0·87	0·47	0·90
Plasma p-tau181 (14/64)	0·92 (0·85 – 0·98)	<0·001	<0·001	5·9 pg/ml	0·93	0·86	0·59	0·98
Plasma GFAP (14/66)	0·84 (0·71 - 0·98)	<0·001	<0·001	174·3 pg/ml	0·86	0·86	0·57	0·97
Age (14/66)	0·77 (0·63-0·91)	0·001	0·002		0·50	0·96	0·70	0·90

AUC: area under curve; CI: confidence interval; p_{FDR}: false discovery rate adjusted p value; PPV: positive predictive value; NPV: negative predictive value. Youden's cut-point is at the coordinates of the ROC curve where a maximum sum of sensitivity and specificity is reached.
